# Evaluation of Ultrasonic Spray Method for Application of Sirolimus-Eluting Coating on Bioresorbable Vascular Scaffolds

**DOI:** 10.3390/ijms26157649

**Published:** 2025-08-07

**Authors:** Katarzyna Jelonek, Joanna Jaworska, Monika Musiał-Kulik, Mateusz Stojko, Jakub Włodarczyk, Michał Sobota, Małgorzata Pastusiak, Anna Smola-Dmochowska, Janusz Szewczenko, Karolina Goldsztajn, Piotr Dobrzyński, Janusz Kasperczyk

**Affiliations:** 1 Centre of Polymer and Carbon Materials, Polish Academy of Sciences, Curie-Sklodowska 34 St., 41-819 Zabrze, Poland; jjaworska@cmpw-pan.pl (J.J.); mmusial@cmpw-pan.pl (M.M.-K.); mstojko@cmpw-pan.pl (M.S.); jwlodarczyk@cmpw-pan.pl (J.W.); msobota@cmpw-pan.pl (M.S.); mpastusiak@cmpw-pan.pl (M.P.); asmola@cmpw-pan.pl (A.S.-D.); p.dobrzynski@ujd.edu.pl (P.D.); 2 Department of Biomaterials and Medical Devices Engineering, Faculty of Biomedical Engineering, Silesian University of Technology, 41-800 Zabrze, Poland; janusz.szewczenko@polsl.pl (J.S.); karolina.goldsztajn@polsl.pl (K.G.); 3 Faculty of Science and Technology, Jan Dlugosz University in Czestochowa, Armii Krajowej Av., 42-200 Czestochowa, Poland; 4 Department of Biopharmacy, Faculty of Pharmaceutical Sciences in Sosnowiec, Medical University of Silesia, Katowice, Jedności 8, 41-200 Sosnowiec, Poland

**Keywords:** biodegradable vascular scaffolds, sirolimus, stent coating, ultrasonic spray coating, restenosis

## Abstract

Restenosis is the main cause of failure after stent implantation during angioplasty. The localized, sustained delivery of an antirestenotic drug may reduce smooth muscle cell (SMCs) proliferation and thereby limit neointimal hyperplasia. The aim of this study was to develop degradable sirolimus-eluting polymer coatings that can be applied on bioresorbable polymer-based scaffolds via an ultrasonic coating system. This is a novel approach because the detailed analysis of the coating procedure on bioresorbable polymeric scaffolds with the use of an ultrasonic system has not been reported thus far. It has been observed that the ultrasonic technique facilitates formation of a smooth coating, well-integrated with the scaffold. However, the drug dose is affected by the concentration of the coating solution and the number of layers. Therefore, these parameters can be used for tailoring the drug dose and release process. Although all types of the developed coatings provided sirolimus elution for at least 3 months, a more uniform, diffusion-controlled release profile was observed from coatings obtained from the 1.0% polymeric solution. The released drug showed antiproliferative activity against vascular SMCs, without any hemolytic or thrombogenic effects. The results of the study may be advantageous for further progress in the development and medical translation of polymeric vascular scaffolds with antirestenotic activity.

## 1. Introduction

Coronary artery disease (CAD) arising from atherosclerosis remains the world’s leading cause of death [1,2]. Percutaneous coronary intervention (PCI), using an expandable coronary stent placed in the affected artery, is currently the primary treatment for cardiovascular diseases [3,4]. A stent’s primary purpose is to serve as a scaffold that restores luminal patency and normal blood flow in blocked atherosclerotic vessels while functioning as a localized delivery system for therapeutic antiproliferative agents. Therefore, implanting drug-eluting stents (DESs) has become the gold standard in PCI because of their efficacy and ability to prevent neointimal proliferation. [5]. The current DESs demonstrated superior clinical performance compared to that of first-generation DESs, with significant reductions in both repeat revascularization procedures and stent thrombosis [6]. The most commonly used antiproliferative agents released from DESs involve sirolimus and its analogues (e.g., everolimus, zotarolimus, biolimus, and novolimus). Sirolimus is a potent immunomodulator that inhibits the mammalian target of rapamycin in smooth muscle cells (SMCs), thereby preventing in-stent restenosis (ISR) [2,4,7,8,9]. Restenosis after PCI is characterized by immediate platelet aggregation and thrombus formation, followed by inflammatory cell infiltration, release of growth factors and cytokines, medial SMCs proliferation and migration into the neointima, and progressive extracellular matrix remodeling [10].

Novel fully bioresorbable vascular scaffolds (BRS) have been designed to overcome the complications of metallic DES, such as hypersensitivity, inflammation, or thrombosis [5]. The bioerodible stents should perform their intended function and gradually degrade over time, restoring natural arterial vasomotion [11]. However, many studies have shown that the side effects of DES and BRS, e.g., inflammation, late thrombosis, and late restenosis, still remain [12,13]. The main reasons of these side effects may be the lack of capacity for adjusting the drug dose and inadequate release behavior [14]. Therefore, there is a need for improvement of drug elution and its better control in the second-generation DESs [4]. For this purpose, the biodegradable drug-eluting coatings, which play a role as a carrier for antiproliferative drugs, are widely explored [15]. The polymer coatings of stents are considered one of the key factors that lead to adverse cardiac events after stent implantation [16], so the development of coatings that show the proper release profile and antirestenotic drug rate is crucial, but very challenging, factor. The rapid release of drugs from the surface of the scaffold may expose the artery to high drug doses, resulting in increased systemic drug levels or delayed re-endothelialization and coverage of the stent struts. In order to improve the efficiency of BRS and control the drug release profile, biocompatible and biodegradable synthetic or natural polymers were used as surface coatings [17]. 

One of the important factors influencing the properties of the antirestenotic coatings is the technological method used for the deposition of a drug-eluting layer. The technical difficulty of coating arises from the need to precisely control both the coating mass and the fine surface quality on the stent, which possesses a micron-scale and highly complex structure [18]. There are various techniques applied for stent coating, e.g., dip coating, electrotreated coating, plasma-treated coating, and spray coating. Ultrasonic atomization is one of the most commonly utilized stent coating techniques. However, the results reported thus far regarding the use of this method refer to metal stents [18,19,20,21]. The scaffold should be considered as a delivery system. Understanding the factors controlling drug release from various delivery systems has been of interest to scientists for many years [22,23,24,25], and it is already well known that various factors may affect the kinetics of drug release, e.g., the properties of the drug delivery system, properties of the polymer, and drug and environmental factors [26]. Thus, unfortunately, the coating methods developed for metal stents cannot be directly used for polymeric scaffolds. In fact, our previous study revealed that the type of stent has a significant impact on the coating system, as differences were even observed for two kinds of polymeric scaffolds obtained from various types of polymers. We observed that using the same composition of the coating layers and the same coating method results in differences in the drug release profile if different types of polymers were used for scaffold’s formation. Scaffolds composed of polymers with higher lactide content, higher molar mass, and higher glass transition temperature caused a slower and bi-phasic erosion-controlled release of sirolimus. In contrast, scaffolds composed of polymers with a lower lactide content provided mainly diffusion-controlled release of sirolimus. The study clearly showed that the scaffold characteristics are crucial factors that must be considered in the development of BRS [27].

A novel approach would be the development of antirestenotic coatings obtained using ultrasonic spray technology that is dedicated for bioresorbable polymeric scaffolds. Therefore, the aim of this study was to develop bioresorbable sirolimus-eluting polymer coatings that can be applied to bioresorbable polymer-based scaffolds using an ultrasonic coating system and to identify parameters for tailoring drug dose and release kinetics. Poly(L-lactide-co-trimethylene carbonate) (PLLA/TMC) was used to form the coatings. Poly(L-lactide) (PLLA) is a semicrystalline biodegradable polymer that undergoes hydrolytic degradation through random scission of ester bonds via a bulk erosion mechanism [28]. Copolymerization has been extensively employed as a strategic approach to precisely tailor material properties [29]. Co-polymers of lactide and TMC are interesting materials for medical applications because they are biodegradable, and poly(trimethylene carbonate) (PTMC) degrades to non-acidic products [30]. Developing a functional and easily applicable coating that releases antirestenotic drugs is crucial for the successful development of bioresorbable vascular scaffolds. Therefore, this study’s results may be valuable for providing further improvements in the field.

## 2. Results

### 2.1. Morphology of Scaffolds

**Figure 1:**
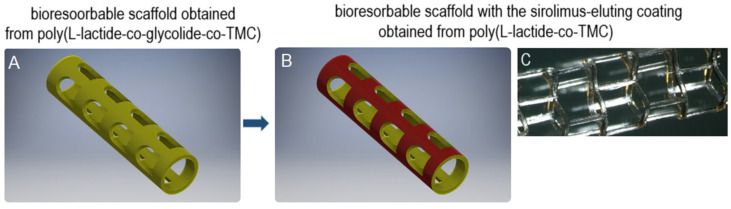
Schematic presentation of bioresorbable scaffolds before (**A**) and after (**B**) application of sirolimus-eluting coating. Optical microscope image of scaffold coated with polymer solution containing sirolimus via the ultrasonic technique (**C**).

The bioresorbable sirolimus-eluting polymer coatings were applied on bioresorbable polymer-based scaffolds via an ultrasonic coating system, as presented in Figure 1.

**Figure 2:**
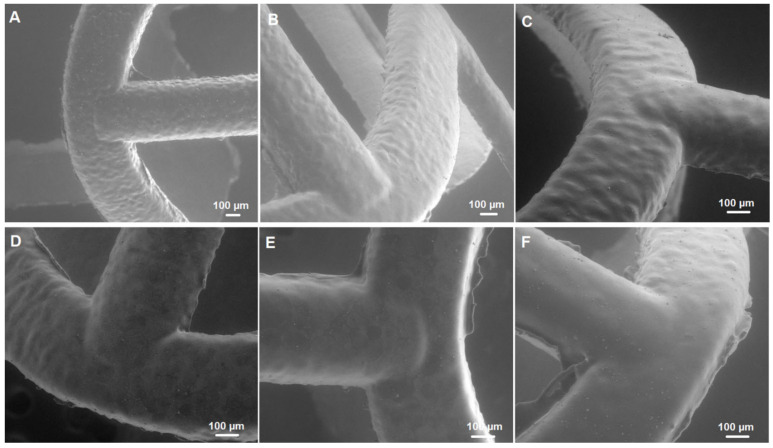
SEM images of coating of scaffolds composed of 3 layers of 1.0% polymer solution (**A**), 5 layers of 1.0% polymer solution (**B**), 7 layers of 1.0% polymer solution (**C**), 3 layers of 2.5% polymer solution (**D**), 5 layers of 2.5% polymer solution (**E**), and 7 layers of 2.5% polymer solution (**F**).

**Figure 3:**
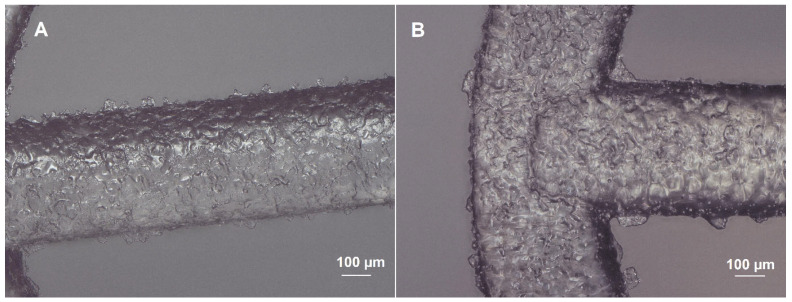
Optical microscope image of coating-free vascular scaffold (**A**) and scaffold coated with 7 layers of 2.5% polymer solution with sirolimus (**B**).

The surface of the bioresorbable cardiovascular scaffolds modified with a biodegradable polymer coating containing an antirestenotic drug via an ultrasonic coating system was analyzed microscopically (Figure 2 and Figure 3). As shown in SEM images, the surface of the stents did not differ, regardless the number of layers and the concentration of the polymer solution (Figure 2). Also, a comparison of the coating-free vascular scaffold (Figure 3A) and the scaffold with the highest drug content obtained using 7 layers of 2.5% polymer solution with the drug (Figure 3B) shows no differences. The struts of both types of scaffolds display a similar appearance and are transparent.

### 2.2. Drug Dose

The analysis of the drug dose obtained after the coating procedure using a different number of layers (3, 5 and 7) and concentration of coating solution (1.0% and 2.5%) was conducted (Table 1). 

The coatings composed of 3 layers obtained from 1.0% polymer solution contained 51 µg of sirolimus. Drug content increased after application of additional layers. The coatings obtained from 2.5% polymer solution contained a significantly higher dose of sirolimus in 3 layers (≈194 µg) compared to the coating obtained from 1.0% polymer solution, and the amount of drug increased in coatings composed of 5 and 7 layers. 

### 2.3. Drug Release 

**Figure 4:**
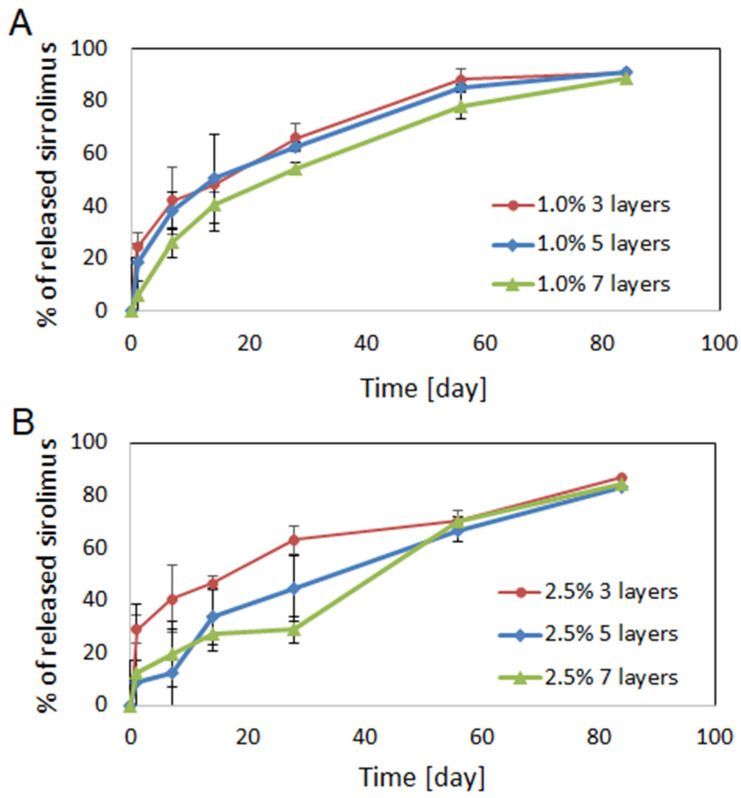
In vitro release of sirolimus from coatings of the bioresorbable scaffolds composed of 3, 5 or 7 layers and obtained from 1.0% (**A**) or 2.5% (**B**) polymer solution (n = 3; ±SD).

The in vitro drug release was evaluated, and the results are presented in Figure 4. 

The profile of drug release from coatings obtained from 1.0% polymer solution did not depend significantly on the number of layers, although scaffold coated with 7 layers characterized slightly slower drug release compared to those for the scaffolds coated with 3 and 5 layers (Figure 4A). The release during 84 days proceeded regularly, without any inhibition periods or rapid burst of drug release. The biggest differences were observed after 1 day of scaffold incubation in PBS, as 24% of drug was released from coatings having 3 layers, 18% from of 5 layers, and only 6% from 7 layers. After 28 days, 66%, 62% and 54% of sirolimus was released from coatings composed of 3, 5 and 7 layers, respectively. Most of drug was released from all types of the coatings obtained from 1.0% polymer solution after 84 days (≈90%).

The release profile of sirolimus from coatings obtained with the use of 2.5% polymer solution depended on the numbers of layers (Figure 4B). Drug release from coatings composed of 3 layers was similar to the release obtained for 1.0% polymer solution and proceeded the most evenly. In the case of 5 and 7 layers, triphasic release was observed. In the first phase, a rapid release of 28% of the drug from coatings composed of 3 layers and ≈12% from 5 and 7 layers was observed. The second phase lasted from 1 to 14 days, or between 1 and 28 days in the case of coatings composed of 5 layers and 7 layers, respectively. This phase was characterized by slower drug release and was followed by increased release of sirolimus in the third phase. Similarly to the layers obtained from 1.0% polymer solution, around 90% of the drug was released from all types of coatings after 84 days. 

The data presented in Figure 4 were fitted to Higuchi and Korsmeyer–Peppas kinetic models to determine the mechanism of sirolimus release (Table 2). It has been observed that the release data present good correlation with the Krosmeyer–Peppas model. In both kinds of coatings (1.0% and 2.5%), the increase in the value of exponent (*n*) is observed with the addition of drug-eluting layers, so the *n* is the lowest for coating composed of 3 layers and the highest for coatings composed of 7 layers. The *n* exponent characterizes the release mechanism, which can be Fickian diffusion (0.5), anomalous transport (0.5 < *n* < 1.0), Case II transport (1.0), or Super Case II transport (higher than 1.0) [31]. In the case of coatings obtained from the 1.0% polymer concentration, the *n* value increases with the number of layers, but for none of them does exceed 0.5, which indicates that the drug release is mainly controlled by diffusion. In the case of the coatings obtained from the 2.5% polymer concentration, the *n* value is below 0.5 only for coating composed of 3 layers and is above 0.5 in the case of coatings composed of 5 and 7 layers, which shows the greater influence of polymer degradation on the drug release process. 

### 2.4. Cell Culture Study

**Figure 5:**
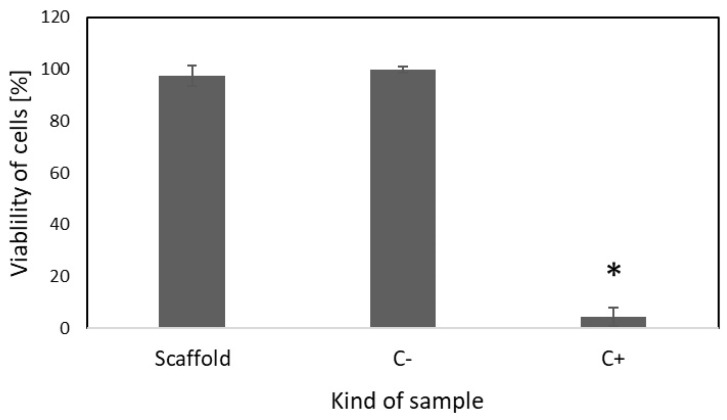
The effect of drug-free scaffold on viability of L-929 cells compared to unmodified cell culture (C-). Medium with dimethyl sulfoxide (DMSO) was used as positive control (C+) (±SD, * *p* < 0.05).

The in vitro cytocompatibility of drug-free scaffolds was evaluated according to ISO 10993-5 and ISO 10993-12 standards via the extraction method with the use of L-929 cells. As presented in Figure 5, the viability of cells incubated in the presence of the extract obtained after incubation of the drug-free scaffold was similar to the viability of the untreated cells. This confirms that the developed scaffolds do not exhibit any cytotoxic effects. 

**Figure 6:**
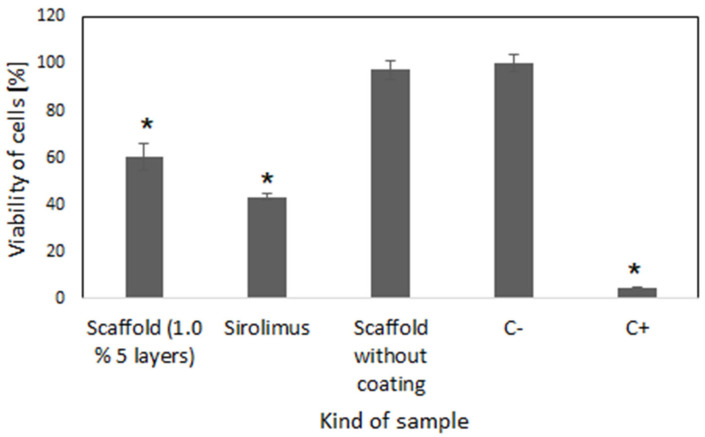
The effect of scaffold coated with 5 layers (1.0%) of coating containing sirolimus, pure drug (sirolimus), and scaffold without coating on viability of HCASMC cells compared to unmodified cell culture (C-). Medium with DMSO was used as positive control (C+) (±SD, * *p* < 0.05).

The cytotoxic activity of the scaffolds with a sirolimus eluting coating composed of 3 layers and obtained from 1.0% polymer was studied against the human coronary artery smooth muscle cells (HCASMC). As shown in Figure 6, the cell growth in the presence of the extract was significantly reduced compared to that in the untreated cells. 

### 2.5. Hemolytic and Thrombogenic Effect

**Figure 7:**
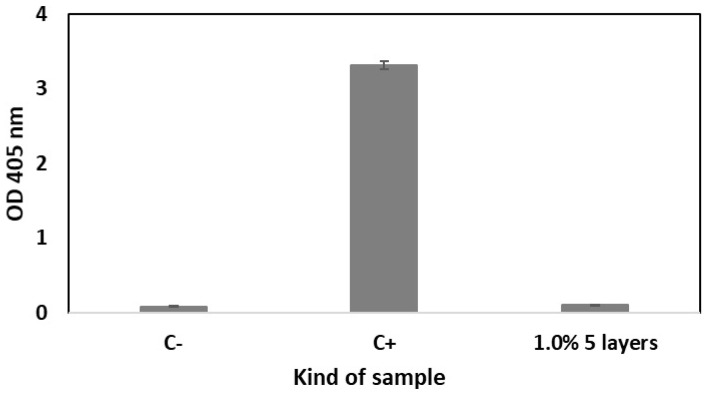
Hemolytic effect of scaffolds coated with 5 layers of 1.0% polymeric solution containing sirolimus compared to results for PBS pH~7 (C-) and 10% Triton X-100 (C+), (±SD, *n* = 3).

Hemolysis is defined as rupturing the membrane of the erythrocytes, causing the release of hemoglobin and other internal components into the surrounding fluid [32]. Hemolytic assay was conducted to evaluate the hemocompatibility of the scaffolds coated with 5 layers of 1.0% polymeric solution containing sirolimus. According to the obtained results, the developed scaffolds did not show any hemolytic effect (Figure 7). 

**Figure 8:**
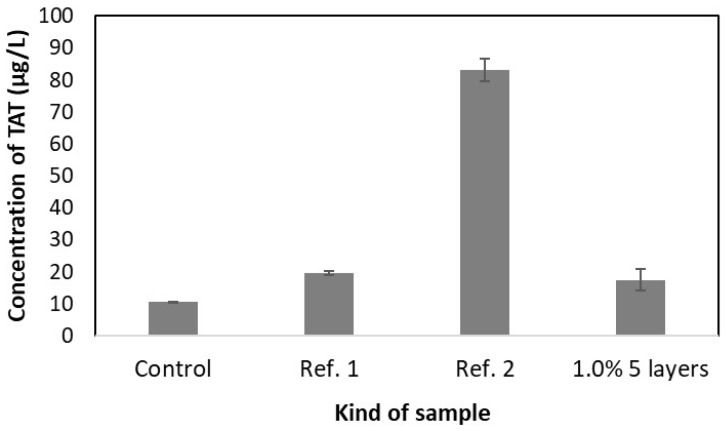
Comparison of concentration of thrombin–antithrombin complex (TAT) determined for blood incubated without sample (control), low-density polyethylene (LDPE) (Ref. 1), medical steel (Ref. 2) and scaffold with 5 layers (1.0%) of coating containing sirolimus (±SD, *n* = 3).

Thrombogenicity measurements included thrombin generation assessed through thrombin–antithrombin complex (TAT) analysis in blood samples following static incubation testing. TAT serves as a widely recognized biomarker of coagulation cascade activation that directly correlates with thrombin concentration in the blood and subsequent fibrin mesh formation [33,34,35]. As shown in Figure 8, the TAT concentration in blood incubated with scaffolds composed of 5 layers of coatings (1%) with sirolimus was insignificant (17.5 µg/L) compared to the levels for blood incubated without any sample (10.5 µg/L) and low-density polyethylene (LDPE), used as a minimally reactive reference (19.7 µg/L). In contrast, the concentration for the medical steel used as a highly reactive reference was significantly increased (83.1 µg/L).

## 3. Discussion

Despite the favorable antiproliferative properties of drug-eluting stents (DESs), in-stent restenosis (ISR) remains a significant clinical concern. [12,36]. The optimized drug dose and release kinetics are expected to prevent vascular SMCs proliferation, while preserving the re-endothelialization process. Nonetheless, achieving controlled, fractional drug release from biodegradable polymeric systems remains challenging because multiple factors—including the polymer and drug properties, coating technique and thickness, coating pore size, release conditions, and post-implantation hemodynamics—influence the release profile [37,38]. The potential disadvantages may be reduced by the proper selection of drugs and polymers [39]. The scaffolds used in our study were obtained from poly(lactide-co-glycolide-co-TMC) [27]. PLA-based polymers represent a promising class of materials for the development of fully resorbable stents [3,39]. The homopolymer of lactide or its copolymers with caprolactone or glycolide is the type most often used as a coating layer for coronary stents [3,40]. We have selected the PLLA/TMC for coating the scaffolds due to promising previous results, which showed its applicability for poly(lactide-co-glycolide-co-TMC) scaffolds [27]. Comparison of the drug release from scaffolds obtained from different biodegradable polymers, but coated with the same kind of sirolimus-eluting layer, demonstrated that sirolimus release from scaffolds composed of polymers with a lower lactide content was faster and mainly diffusion-controlled. In contrast, the scaffold composed of polymers with a higher lactide content showed a slower and bi-phasic, erosion-controlled release of sirolimus. This effect was likely caused by the high penetration of the drug into the scaffold (facilitated by the dip coating method, which was applied for stent coating), resulting in the fact that not only the coating but also the polymer forming the scaffold controlled the drug release process [27]. In the current study, ultrasonic atomization was used to form the drug eluting layer, which is not expected to facilitate penetration of the drug into the scaffold. Generally, there are several coating techniques offering their own benefits and drawbacks, e.g., dip coating, electro-treating, plasma-treating, and spray coating (ultrasonic atomization, electrohydrodynamic jetting, and air-brush spray coating). Spray coating enables the tailoring of the coating parameters, resulting in better optimization of the release profile [19]. In fact, using different parameters of ultrasonic atomization, i.e., different numbers of layers (3, 5, and 7) or concentration of polymer solution (1% and 2.5%) allowed for obtaining various drug doses (Table 1), release rates, and profiles (Figure 4). The influence of both factors, i.e., the concentration of coating solution and number of layers, on drug dose was observed, which can be used as parameters for tailoring the drug dose and release profile. The amount of drug increased significantly in coating layers produced from 2.5% polymer solution compared to those using 1.0% polymer solution. Also, an increased drug amount may be obtained via the addition of coating layers. The duration of drug release from the studied coatings is similar to that of the bioresorbable coatings of the commercially available stents (Figure 4). The Oshiro stent, obtained from cobalt–chromium alloy and coated with PLLA, is characterized by the release of 50% of the drug in 30 days and 80% of the drug in 3 months [40]. In fact, the drug release period from currently available polymer coatings of DESs differs from less than 1 month (BioFreedom), 3–4 months (Xience, Orsiro, Ultimaster, Synergy), 6 months (Onyx), or even 9 months (MiStent) [4,41]. However, the ideal kinetics of drug release for DESs is currently unknown [17]. It is believed that vascular SMCs start to hyperproliferate after 24 hours of stent implantation, and this continues for around 2 weeks. Therefore, antirestenotic drugs need to be delivered for at least 3 weeks after stent implantation to prevent SMCs proliferation and migration into the stent [19]. In the case of the developed sirolimus-eluting coatings, more uniform drug release was observed from coatings obtained from 1.0% polymeric solution (Figure 4). Moreover, analysis of the mechanism of drug release (Table 2) revealed that the coatings obtained from the 1.0% polymer solution released drugs mainly by diffusion. In the case of the coatings obtained from the 2.5% polymer solution, only coating composed of 3 layers demonstrated diffusion-controlled sirolimus release. In coatings composed of 5 and 7 layers, a higher influence of polymer degradation on the drug release process was observed. Generally, in both cases, i.e., the 1.0% and 2.5% solutions, the increase in coefficient *n* value was observed with the addition of layers. Thus, the diffusion-controlled drug release is the most significant from coatings composed of the smallest number of layers. The addition of layers causes an increase in the influence of the polymer on drug release. These observations suggest that there may be a higher penetration of the drug into the scaffold in the case of coatings comprised of higher numbers of layers. 

Therefore, the scaffolds containing 5 layers with sirolimus obtained from 1.0% polymeric solution were selected based on the advantageous uniform drug release profile and average drug release amount for analysis of their antiproliferative activity and compatibility with blood. Also, the biological evaluation was conducted for drug-free scaffolds to confirm their cytocompatibility (Figure 5). The viability of cells was evaluated based on the CCK-8 assay, which is a sensitive colorimetric technique for the determination of the number of viable cells. The WST-8 (2-(2-methoxy-4-nitrophenyl)-3-(4-nitrophenyl)-5-(2,4-disulfophenyl)-2H tetrazolium, monosodium salt) is reduced by cellular dehydrogenases to an orange formazan product. The number of living cells is directly proportional to the amount of the produced formazan. The scaffolds containing 5 layers of coatings eluting sirolimus caused significant decrease in the proliferation of HCASMC (Figure 5). Over-proliferation of the vascular SMCs is related to restenosis [41]. It has been determined that during the late phase after stent implantation, the vascular SMCs, normally highly differentiated, change their phenotype, become hypertrophic, and then proliferate and migrate at a high rate. This aberrant vascular SMCs growth associated with coordinated extracellular matrix synthesis leads to neointima hyperplasia and in-stent restenosis [42]. Finally, the lack of hemolytic effect (Figure 7) and thrombogenicity (Figure 8), confirmed under an in vitro environment, show the potential of the scaffolds created with the developed sirolimus-eluting coating. Future studies should focus on the analysis of their in vivo applicability. 

## 4. Materials and Methods

### 4.1. Materials

The polymers were synthesized at the Centre of Polymer and Carbon Materials, Polish Academy of Sciences, Zabrze, Poland. Sirolimus (rapamycin) was purchased from LC Laboratories (Woburn, USA). All other reagents and organic solvents of analytic grade were purchased from Sigma-Aldrich (Poznań, Poland). Poly(lactide-co-glycolide-co-trimethylene carbonate) (PLLA-GA-TMC) was used for formation of cardiovascular scaffolds by microinjection molding, and it was synthesized according to the previously published data [43]. Poly(L-lactide-co-trimethylene carbonate) (PLLA/TMC) was used for preparation of the coating. The polymerization of PLLA/TMC was performed according to the methods of Refs. [27,44].

### 4.2. Coating Procedure

Cardiovascular scaffolds (length, 15 mm; Ø, 5.4 mm) were obtained from poly(lactide-co-glycolide-co-trimethylene carbonate) (PLLA/GA/TMC) via microinjection molding. The PLLA/TMC, characterized by a lactide to TMC molar ratio of 75:25, a molar mass (M_n_) of 49 kDa, and a glass transition temperature (T_g_) ≈ 35 °C [28], was used to form the coatings. The detailed characteristics of the physicochemical properties of the PLLA/TMC has been described in our previous manuscript [27]. The scaffolds were coated with a sirolimus eluting layer via an ultrasonic method using ExactaCoat (Sono-Tek; New York, NY, USA), equipped with a 120 kHz nozzle, using impact ultrasonic spray shaping. The preparation of the coating solution was as follows: PLLA/TMC with sirolimus (with a polymer to drug ratio of 4:1 *w/w*) was dissolved in methylene chloride to obtain a 1.0% or 2.5% (*w/w*) solution. The drug-free solution was prepared for comparison. To study the factors influencing the properties of the coating, the following parameters were compared: concentration of polymer solution (1.0% *vs.* 2.5%) and number of layers (3, 5 and 7). The drying of the coated scaffolds was conducted for 7 days at reduced pressure (105 mbar).

### 4.3. Characterization of Scaffold Morphology

The scaffold morphology was analyzed by means of scanning electron microscopy (SEM; Quanta 250 FEG, FEI Company, Hillsboro, OR, USA) and optical microscope (KEYENCE, VHX 7000, Itasca, IL, USA). SEM analysis were conducted under low vacuum conditions (80 Pa) at an acceleration voltage of 5 kV from secondary electrons collected by a large-field detector.

### 4.4. Evaluation of Drug Dose and In Vitro Release

The drug dose in the scaffolds and the amount of sirolimus released was determined by means of high-performance liquid chromatography (HPLC). A LiChrospher^®^ 100 RP-18 LiChroCART^®^ 250–4 mm (5 μm) column and a LiChrospher® 100 RP-18 LiChroCART® 4–4 mm (5 μm) guard column were maintained at 40 °C. Methanol and 0.1% formic acid (85:15 *v/v*) was used as the mobile phase (flow rate of 1 mL/min). The injection volume was 20 μL. The analyte was traced at the maximum absorbance (278 nm). The linear calibration curve of sirolimus was in the range of 0.5–500 μg/mL, with correlation coefficient R^2^ = 0.9995.

The extraction method was used for evaluation of the drug dose [45,46]. Briefly, the scaffolds were dissolved in of methylene chloride (0.5 mL) and stirred at 500 rpm for 30 min. The drug was extracted via the addition of ethanol (2.5 mL), and the samples were stirred for another 30 min. Then, the samples were centrifuged (3500 rpm for 10 min) and analyzed using HPLC.

Analysis of in vitro drug release was conducted at 37 °C in 5 mL of phosphate-buffered saline (PBS) at pH 7.4 under constant agitation (120 rpm). The experiment was conducted in triplicate. The PBS was replaced after 1, 3, and 7 days and then once a week thereafter. After 1, 3, 7, 14, 28, 56, and 84 days of incubation, 3 samples were collected for evaluation of the released drug by means of the extraction method. The modeling of the kinetics of the drug release process was conducted by fitting the release profiles with time-dependent equations using the DDsolver, an Excel add-in program [47]. The following mathematic models were applied to determine the drug release rates from the scaffolds, i.e., Higuchi and Korsmeyer–Peppas models. The Higuchi model is described by the following expression: Mt = KH∙t½, where Mt is the amount of drug release at time t, KH is the Higuchi dissolution constant, and k is the rate constant. The Korsmeyer–Peppas equation is Mt/M∞ = Kkptn, where Mt/M∞ is the fraction of drug release at time t, Mt is the amount of drug released in time t, M∞ is the amount of drug released after time ∞, Kkp is the Korsmeyer release rate constant, and *n* is the diffusional exponent or drug release exponent [31,48]. The best fitting model is based on R^2^ (coefficient of determination).

### 4.5. Cytotoxicity Study

The cytocompatibility of the drug-free scaffold was analyzed in vitro according to the ISO 10993-5 and ISO 10993-12 standards with the use of the L-929 mouse fibroblast cell line (CCL-1 ^™^, ATTC) and the elution test method. Cells were cultured in Eagle’s Minimum Essential Medium supplemented with 100 U/mL penicillin, 100 μg/mL streptomycin, and 10% fetal bovine serum. The 10 mM HEPES was used in the experimental cell culture. Fibroblasts were maintained at 37 °C in a humidified atmosphere containing 5% CO_2_ (Incubator Eppendorf Cell Expert). The extracts were obtained by placing the scaffolds in cell culture media at 37 °C for 24 h. Then, the scaffolds were removed, and the extracts were used in cell culture study. L-929 cells were seeded in 96-well plates at a density of 4 × 10^3^ cells per well and cultured for 24 h under standard conditions in a CO_2_ incubator to allow for cell attachment. Then, the culture medium was replaced with the previously prepared extracts and incubated for 72 h. Untreated cells were used as a negative control (C-), and cells treated with 5% DMSO were used as a positive control (C+). The viability of cells was conducted with the use of a Cell Counting Kit-8 (CCK-8) assay. According to the protocol of the assay, the absorbance was read at 450 nm and 650 nm (reference wavelength) using Spark 10M (Tecan, Männedorf, Switzerland). 

The antiproliferative properties of the scaffolds coated with sirolimus (by using 5 layers of 1.0% polymer solution) were analyzed via the elution test method with the use of the primary human coronary artery smooth muscle cells (HCASMC) (PCS-100-021^™^, ATCC). The cells were grown in the Vascular Cell Basal Medium, supplemented with Vascular Smooth Muscle Growth Kit Components (rh FGF-basic, 5 ng/mL; rh insulin, 5 µg/mL; ascorbic acid, 50 µg/mL; L-glutamine, 10 mM; rh EGF, 5 ng/mL; Fetal Bovine Serum, 5%), 20 U/mL penicillin, and 20 μg/mL streptomycin. The cells were maintained at 37 °C in a humidified atmosphere containing 5% CO_2_. The extracts were obtained using the same procedure as described above for the drug-free scaffolds. The HCASMC cells were seeded in 96-well plates at a density of 4 × 10^3^ cells per well and cultured for 24 h. Then, the culture medium was replaced with the previously prepared extracts and incubated for 72 h. For comparison, the sirolimus solution was used at a concentration of 20 µg/mL. Untreated cells were used as a negative control (C-), and cells treated with 5% DMSO were used as a positive control (C+). The viability of cells was determined with the use of the CCK-8 assay. 

The cell culture experiments were conducted twice, using at least eight repetitions. For statistical analysis of the results, one-way ANOVA was used.

### 4.6. Compatibility with Blood

Hemocompatibility evaluations for the selected sample (scaffolds coated with sirolimus by using 5 layers of 1.0% polymer solution) were performed according to ISO 10993-4. Human blood was obtained from healthy volunteer donors who had not used medications in the previous 2 weeks (approval of Bioethical Commission of the Medical University of Silesia in Katowice, Poland: BNW/NWN/0052/KB1/25/24 on 9 April 2024). Freshly drawn blood (not older than 30 min) was used in all experiments. 

*Hemolysis Assay*

The procedure described by Sæbø et al., with some modifications, was used for evaluation of the hemolysis [49]. The blood collected from healthy volunteers in lithium heparin tubes (IMPROVE, Lot A62010; 4 mL) was centrifuged at 1700× *g* for 5 min. The erythrocytes were washed three times via the addition of PBS pH~7 (2 mL) and centrifugation. The scaffolds were incubated at 37 °C for 60 min in 1 mL of 1% erythrocyte suspension. As a positive control (C+), a 10% Triton X-100 was used, and PBS pH~7 was employed as a negative control (C-). After centrifugation, the absorption of the supernatant was measured at 405 nm in a plate reader (Tecan; Männedorf, Switzerland).

*Static Blood Incubation Test*

A static incubation test was conducted according to the procedure described in the literature [34,35,50]. Human blood was drawn in 10 mL no-additive Improvacutainer^®^ tubes (IMPROVE, Ref. 604100200, 10 mL) supplemented with 1 mL of 5 IU/mL heparin (Heparium WZF, 5000 I.E./5 ml, Polfa Warszawa S.A., Poland) to reach a final concentration of 0.5 IU heparin per 1 mL of blood. For static blood incubation, samples were placed in cryotubes (CryoKING, 5 mL vials) with 1 mL of blood. Incubation was performed at 37 °C for 1 h, and the cryotubes were reversed every 10 min to avoid cell sedimentation. Low-density polyethylene (LDPE) was used as a minimally reactive reference (Ref. 1), and medical steel was employed as a highly reactive reference (Ref. 2). Blood without sample was used as a control (Control). After incubation, blood samples were introduced into 3.2% sodium citrate tubes (VACUETTE, Ref. 455322, Lot A240435H; 9 mL) and immediately centrifuged at 2500 g for 20 min at 4 °C; then blood plasma was collected and stored at −80 °C until further analysis using immunosorbent assays (ELISA). Activation of the coagulation cascade was quantified by evaluation of the concentration of thrombin–antithrombin complex (TAT) (Enzygnost^®^ TAT micro assay; Siemens Healthineers, Erlangen, Germany).

### 4.7. Statistical Analysis

All the experiments were conducted at least twice using 3 samples in the case of drug release study and the evaluation of cytocompatibility with the drug, or at least 8 samples in each experiment in the case of cytotoxicity study. For statistical analysis of the results, one-way ANOVA was used with the post hoc Tukey test. The homogeneity of variance was checked using Levene’s test. All the results are expressed as means ± SD. The *p* value of <0.05 was considered statistically significant.

## 5. Conclusions

The analysis of the parameters of the ultrasonic coating procedure for the bioresorbable scaffolds was conducted. The ultrasonic method enabled the formation of a smooth coating composed of sirolimus-containing PLLA/TMC, which was well-integrated with the scaffold. The morphology of the scaffolds did not change after application of the coating, regardless of the number of layers and the concentration of the polymer solution. The study showed that both 1.0% and 2.5% polymer solutions are appropriate for ultrasonic coatings. It has been determined that the drug content may be adjusted by varying the number of layers and the concentration of polymer solution. More uniform drug release, controlled mainly by diffusion, was observed for coatings obtained from the 1.0% polymeric solution compared to the results for the 2.5% polymeric solution. All types of the developed coatings provided sirolimus release for at least 3 months. Moreover, the released drug showed antiproliferative activity against vascular SMCs, along with a lack of hemolytic and thrombogenic effects. The results of the study may be valuable for the further development and clinical translation of polymeric vascular scaffolds with antirestenotic activity. 

## Figures and Tables

**Table 1:** Comparison of drug dose in coatings of the bioresorbable scaffolds composed of 3, 5 or 7 layers and obtained from 1.0% or 2.5% polymer solution (*n* = 5; ±SD).

Concentration of Coating Solution	Sirolimus Content in Coating of Scaffold (µg)		
	3 Layers	5 Layers	7 Layers
**1.0%**	51.0 ± 0.7	100.3 ± 10.3	143.1 ± 5.6
**2.5%**	193.7 ± 15.8	300.1 ± 0.7	415.3 ± 17.9

**Table 2:** Model parameters of sirolimus release from scaffolds.

Model	Kind of Sample					
	1.0% 3 Layers	1.0% 5 Layers	1.0% 7 Layers	2.5% 3 Layers	2.5% 5 Layers	2.5% 7 Layers
**Higuchi** R^2^	0.9158	0.9499	0.9936	0.8481	0.9781	0.9315
**Korsmeyer–Peppas** R^2^ *n*	0.9897 0.326	0.9963 0.357	0.9938 0.490	0.9851 0.278	0.9876 0.590	0.9575 0.676

## Data Availability

The data that support the findings of this study are available from the corresponding author upon reasonable request.
